# Cardiac Cell Therapy with Pluripotent Stem Cell-Derived Cardiomyocytes: What Has Been Done and What Remains to Do?

**DOI:** 10.1007/s11886-022-01666-9

**Published:** 2022-03-11

**Authors:** Dinesh Selvakumar, Leila Reyes, James J. H. Chong

**Affiliations:** 1 Centre for Heart Research, The Westmead Institute for Medical Research, The University of Sydney, Westmead, NSW Australia; 2 Department of Cardiology, Westmead Hospital, Westmead, NSW Australia

**Keywords:** Stem cells, Embryonic stems cells, Induced pluripotent stem cells, Cardiomyocytes, Cardiac cell therapy, Heart regeneration

## Abstract

**Purpose of Review:**

Exciting pre-clinical data presents pluripotent stem cell-derived cardiomyocytes (PSC-CM) as a novel therapeutic prospect following myocardial infarction, and worldwide clinical trials are imminent. However, despite notable advances, several challenges remain. Here, we review PSC-CM pre-clinical studies, identifying key translational hurdles. We further discuss cell production and characterization strategies, identifying markers that may help generate cells which overcome these barriers.

**Recent Findings:**

PSC-CMs can robustly repopulate infarcted myocardium with functional, force generating cardiomyocytes. However, current differentiation protocols produce immature and heterogenous cardiomyocytes, creating related issues such as arrhythmogenicity, immunogenicity and poor engraftment. Recent efforts have enhanced our understanding of cardiovascular developmental biology. This knowledge may help implement novel differentiation or gene editing strategies that could overcome these limitations.

**Summary:**

PSC-CMs are an exciting therapeutic prospect. Despite substantial recent advances, limitations of the technology remain. However, with our continued and increasing biological understanding, these issues are addressable, with several worldwide clinical trials anticipated in the coming years.

## Introduction

Myocardial infarction, the most common cause of heart failure, results in the death of up to 1 billion highly specialized cardiomyocytes [1]. The loss of these cells is accentuated by the inability of the adult heart to meaningfully regenerate, resulting in the transformation of contractile heart tissue into akinetic and fibrotic scar [2–4]. Though considerable advances in the treatment of myocardial infarction have now been made, heart failure remains a devastating illness, responsible for great morbidity, mortality and economic burden worldwide [5–7].

In recent years, delivery of exogenous cells has emerged as a favourable therapeutic strategy for the replacement of cardiomyocytes lost after injury. Numerous cell types, predominantly of adult stem cell origin, have now been tested in clinical trials, though results on efficacy of these cells have been mixed [8]. It is now clear that these adult stem cells lack capacity to differentiate into working cardiomyocytes, exerting any beneficial effect through paracrine mechanisms rather than remuscularization [9, 10].

The discovery of the pluripotent stems cells (PSCs), an umbrella term encompassing embryonic stem cells (ESCs) and induced pluripotent stem cells (iPSCs), was a breakthrough in regenerative medicine due to their scalability and capacity to differentiate into virtually all somatic cell types [11, 12]. Since their conception, a plethora of studies have demonstrated the capacity for both ESCs and iPSCs to differentiate into cardiomyocytes, allowing for a near-limitless supply of functional cardiomyocytes for cell therapy applications.

**Fig. 1:**
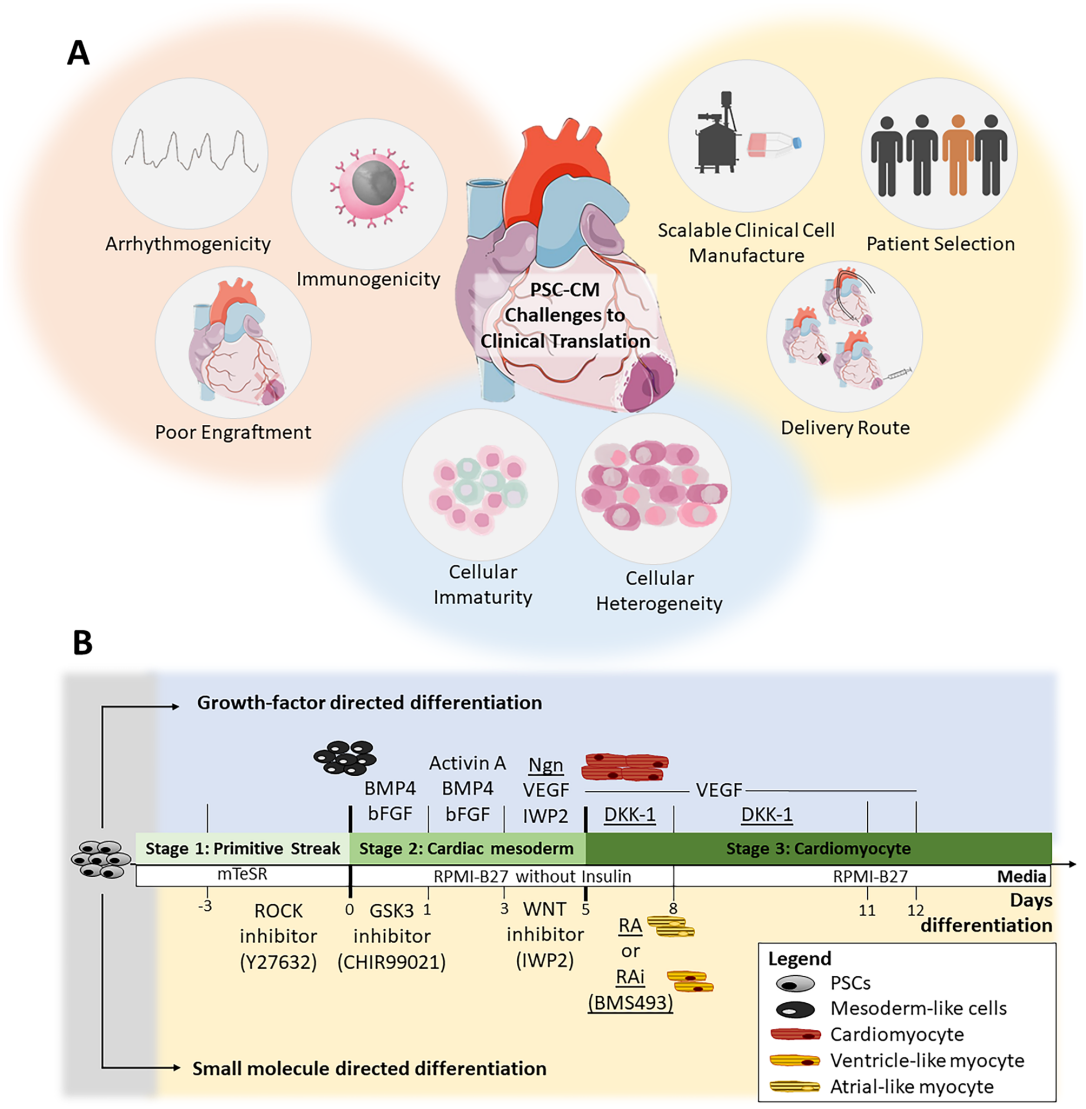
**A** Summary of challenges to PSC-CM clinical translation. **B** Scheme of cardiac differentiation strategies using growth-factors and small molecules. *Underlined agents* are optional additives to promote specific sub-population differentiation. BMP, bone morphogenic progenitor; DKK-1, Dickkopf-1; GSK, glycogen synthase kinase; Ngn, Noggin; IWP, inhibitors of Wnt ligand production; PSC-CM, pluripotent stem cell-derived cardiomyocyte; RA, retinoic acid; Rai, retinoic acid inhibitor; ROCK, Rho-kinase protein kinase; VEGF, vascular endothelial growth factor

Pre-clinical transplantation studies followed soon after, and PSC-CMs have now been shown to remuscularize and improve function in clinically relevant large animal myocardial infarct models (Table 1) [13••, 14••, 15••, 16••]. Interest in this cell type has now grown significantly, and clinical trials around the world are imminent [17–20] (Table 1). However, several translational hurdles have been identified, requiring solutions before this technology can be widely embraced as a serious contender to current clinical approaches (Fig. 1A).

**Table 1:** Important large animal pre-clinical studies and registered clinical trials investigating PSC derivatives

**Pre-clinical studies in large animal models**				
**Study (year)**	**Study cohort**	**Cell dose and delivery route**	**Comments**	**Reference**
**Human embryonic stem cell-derived cardiomyocytes regenerate non-human primate hearts (2014)**	• Pigtail macaques • *n* = 7 ◦ 6 cell-treated ◦ 1 vehicle control • Cells delivered 2 weeks after myocardial infarct creation by percutaneous ischaemia–reperfusion	• 1 billion human ESC-CM • Epicardiallly injected via left thoracotomy	• Established feasibility of large-scale PSC-CM production and cryopreservation for transplantation applications • Demonstrated robust remuscularization capacity of PSC-CM therapy in non-human primate model, with grafts shown to be perfused by and electromechanically coupled with host heart • Post-transplant ventricular arrhythmias noted	[13••]
**Allogeneic transplantation of iPS cell-derived cardiomyocytes regenerates primate hearts (2016)**	• Cynomolgus monkey • *n* = 10 ◦ 5 cell-treated ◦ 5 vehicle control • Cells delivered 2 weeks after myocardial infarct creation by surgical ischaemia–reperfusion	• 400 million MHC-matched allogeneic iPSC-CM • Epicardially injected via sternotomy	• Established efficacy of allogeneic transplantation of iPSC-CM in non-human primate myocardial infarct model • Cell grafts survived up to 12 weeks post transplantation and improved left ventricular function • Post-transplant ventricular arrhythmias noted	[16••]
**Human embryonic stem cell-derived cardiomyocytes restore function in infarcted hearts of non-human primates (2018)**	• Pigtail macaques • *n* = 9 ◦ 5 cell-treated ◦ 4 vehicle control • Cells delivered 2 weeks after myocardial infarct creation by percutaneous ischaemia–reperfusion	• 750 million human ESC-CM • Epicardially injected via left thoracotomy	• Showed transplantation of PSC-CM improves cardiac function post myocardial infarction in non-human primate model (~ 10% absolute LVEF increase after 1 month of therapy) • Post-transplant ventricular arrhythmias noted. Electro-anatomical mapping suggests cell grafts act as ectopic pacemaking focus	[14••]
**Large cardiac muscle patches engineered from human induced-pluripotent stem cell-derived cardiac cells improve recovery from myocardial infarction in swine (2018)**	• Female Yorkshire pigs • *n* = 50 ◦ 13 cell patch ◦ 14 control patch ◦ 15 no patch ◦ 8 sham surgery • Patches delivered immediately following creation of myocardial infarction by surgical ischaemia–reperfusion	• 2 × cell patches consisting of: ◦ 4 million human iPSC-CMs ◦ 2 million human iPSC-endothelial cells ◦ 2 million human iPSC-smooth muscle cells • Epicardially delivered by sternotomy	• Cardiac muscle patches were created with tri-lineage PSC derivatives • Cardiac function improved and infarct size reduced after transplantation of muscle patches in porcine model of acute myocardial infarction • No ventricular arrhythmias were observed	[85]
**Pharmacologic therapy for engraftment arrhythmia induced by transplantation of human cardiomyocytes (2021)**	• Yucatan mini-pigs *• n* = 9 (phase 1) ◦ All cell-treated *• n* = 19 (phase 2) ◦ 9 cell and anti-arrhythmic drug treated ◦ 7 cell-treated ◦ 2 vehicle control • Cells delivered 2 weeks after myocardial infarct creation by percutaneous ischaemia–reperfusion	• 500 million human ESC-CM • First 3 subjects underwent cell transplantation by epicardial injection via sternotomy • Remaining subjects underwent cell transplantation by percutaneous trans-endocardial injection	• Efficacy of clinically available anti-arrhythmic drugs in controlling PSC-CM related engraftment arrhythmia tested • Series of anti-arrhythmics acutely tested in phase 1, with ivabradine and amiodarone shown to be the best performing agents • Chronic treatment with combination of ivabradine and amiodarone tested in phase 2, with drug treatment reducing engraftment arrhythmia rate and burden and improving mortality	[30•]
**Cyclin D2 overexpression enhances the efficacy of human induced pluripotent stem cell-derived cardiomyocytes for myocardial repair in a swine model of myocardial infarction (2021)**	• Female Yorkshire pigs *• n* = 28 ◦ 7 standard PSC-CM ◦ 7 Cyclin D2 overexpressed PSC-CM ◦ 7 vehicle control ◦ 7 sham surgery • Cells delivered immediately following creation of myocardial infarction by surgical ischaemia reperfusion	• 30 million human iPSC-CM • Epicardially injected via sternotomy	• Validation of enhanced proliferative capacity of PSC-CM with cyclin D2 overexpressed • Due to enhanced proliferative capacity, lower cell dose required • No ventricular arrhythmias observed	[51]

**Completed and registered clinical trials**				
**Trial name (year)** **Status—Country** **Official title**	**Study cohort**	**Cell dose and delivery route**	**Comments**	**Reference**
**ESCORT (2018)** **• Completed—France** **• Transplantation of human embryonic stem cell-derived cardiovascular progenitors for severe ischaemic left ventricular dysfunction**	• Severe ischaemic cardiomyopathy (LVEF 15–35%) greater than 6 months post myocardial infarction and with indication for surgical coronary revascularisation *• n* = 6 ◦ All cell-treated	• ~ 8.2 million human ESC-derived cardiovascular progenitor cells embedded in fibrin patch • Epicardially delivered during surgical coronary revascularisation	• Technical feasibility and short- and medium-term safety demonstrated (18-month follow-up) • One patient died early post-operatively from treatment unrelated comorbidities • One patient died of heart failure after 22 months • No arrhythmias or tumour detected in any patients	[22••]
**BioVAT-HF** **• Recruiting—Germany** **• Safety and efficacy of induced pluripotent stem cell-derived engineered human myocardium as biological ventricular assist tissue in terminal heart failure**	• Severe heart failure patients (LVEF < 35%) not eligible for heart transplantation *• n* = 53 ◦ All cell-treated	• Engineered heart muscle patch constructed from mixture of iPSC-CM and iPSC-stromal cells embedded in bovine collagen hydrogel • Surgically implanted by left-lateral mini-thoracotomy or during other clinically indicated intervention (e.g. Valve surgery or coronary artery bypass grafting)	Primary outcome: • Regional function as measured by serial echocardiography and/or cardiac MRI over 12 months	[17]
**HEAL-CHF** **• Recruiting – China** **• Epicardial injection of allogeneic human PSC-derived cardiomyocytes to treat severe chronic heart failure**	• Severe chronic ischaemic cardiomyopathy (LVEF 20–45%) with indications for surgical coronary revascularisation *• n* = 20 ◦ 10 cell-treated ◦ 10 controls	• 200 million human PSC-CMs • Epicardially injected during coronary artery bypass grafting • Control group will receive coronary artery bypass grafting only	Primary outcome: • Incidence of sustained ventricular arrhythmia up to 6 months post-operation • Incidence of newly formed tumours up to 6 months post-operation Secondary outcomes: • Left ventricular function • Functional status • Adverse events • Generation of reactive antibodies • NT-proBNP	[18]
**Treating congestive heart failure patients with human iPSC-derived cardiomyocytes through catheter-based endocardial injection** • **Recruiting – China**	• Severe congestive heart failure (LVEF < 40%) *• n* = 20 ◦ 10 low-dose cell treatment ◦ 10 high-dose cell treatment	• Human iPSC-CM • Low dose: 100 million cells • High dose: 400 million cells • Delivered by transcatheter endocardial injection system	Primary outcome: • Incidence of major serious adverse events in first month post catheterisation Secondary outcome: • Left ventricular function • Functional status • Incidence of severe arrhythmias • Incidence of newly formed tumours • Generation of reactive antibodies	[19]
**LAPiS** **• Registered, not yet recruiting – Japan** **• A phase I/II study of human iPS cell-derived cardiomyocyte spheroids in patients with severe heart failure secondary to ischaemic heart disease**	• Severe ischaemic cardiomyopathy (LVEF < 40%) greater than 1 month post myocardial infarction *• n* = 10 ◦ 5 low dose cell treatment ◦ 5 high dose cell treatment	• Human iPSC-CM spheroids suspension • Low-dose and high-dose strategies to be tested • Exact cell dosages and delivery route not yet described	Primary outcome: • Safety and tolerability within first 26 weeks after transplantation Secondary outcomes: • Left ventricular function • Myocardial blood flow and viability • 6-min walk distance • Quality of life questionnaires • NT-proBNP	[20]

*ESC* Embryonic stem cell, *MHC* major histocompatibility complex, *iPSC* induced pluripotent stem cell, *LVEF* left ventricular ejection fraction, *MRI* magnetic resonance imaging, NT-proBNP N-terminal pro B-type natriuretic peptide, *PSC-CM* pluripotent stem cell-derived cardiomyocyte

In this review, we summarize the pre-clinical work investigating PSC-CM cardiac cell therapy, highlighting limitations that may inform the refinement of cardiomyocyte differentiation strategies. We then outline the various methods used to differentiate cardiomyocytes from PSC cultures and characterize the cell types arising from these conditions. This is done to help inform future strategies which may produce PSC-CMs fit for widescale clinical translation.

## Summary of Pre-clinical Studies

Interest in harnessing the regenerative potential of PSCs or their derivatives has been pursued over the past two decades. Unlike adult stem cells, PSCs are highly expandable and have the capacity to differentiate into virtually all somatic cell types, providing an unlimited source of cardiomyocytes and the prospect of replacing inert myocardial scar with functional muscle graft. Initial speculation that environmental cues could direct undifferentiated PSCs to a cardiomyocyte lineage after transplantation into the heart resulted only in immune rejection or teratoma formation [21].

Attention turned instead toward transplantation of PSC-derived cardiovascular progenitors (CVPs) or bona-fide cardiomyocytes. Of these two products, CVPs have now been evaluated in the clinical arena, with a phase 1 clinical trial demonstrating safe delivery of these cells embedded in a fibrin patch to patients undergoing coronary artery bypass surgery [22••]. The feasibility and safety of this approach was demonstrated, paving the way for larger studies examining efficacy. However, pre-clinical evaluation of this cell type suggests a lack of durable engraftment, with any beneficial effects likely secondary to paracrine mechanisms rather than direct remuscularization [23, 24].

This is contrasted with PSC-CM, which now have robust data proving both remuscularization capacity and cardiac functional improvement post myocardial infarction in small and large animal models [13••, 14••, 16••, 25–29, 30•]. In 2014, Chong et al. [13••] showed remarkable remuscularization capacity of this therapy after transplantation of 1 billion PSC-CMs into the infarcted hearts of pigtail macaques. Grafts were perfused by host vasculature and electromechanically coupled to native myocardium. Follow-up work, also in non-human primate subjects, confirmed the functional benefits of PSC-CM grafts, showing durability of the cell graft and near normalization of left ventricular ejection fraction by three months post-transplant in cell recipients [14••, 16••]. Explanations for this dramatic effect were postulated to be a combination of both cell-mediated paracrine influences as well as direct contractile force generated by the cardiomyocyte graft.

There is now global interest in this cell type, with unpublished reports of first-in-human delivery of this therapy already emerging from China and pilot clinical trials at various stages of development in Japan, North America and Europe [31]. However, the aforementioned pre-clinical studies have also identified several hurdles that must be overcome prior to successful clinical translation. Cardiac cell therapy is exceedingly complex, and a worldwide, cross-disciplinary approach to address mechanistic, clinical and regulatory hurdles is essential to ensure successful translation. The following is a discussion of some of the key clinical challenges faced by this therapy.

## Barriers to Translation

### Immunogenicity

Immune rejection is a major issue affecting the retention and survival of transplanted PSC derivatives [32]. Though undifferentiated PSCs may possess immune privilege properties, expressing low or absent levels of major histocompatibility complex (MHC) antigens and co-stimulatory molecules, increased MHC expression has been documented after differentiation leading to detection of the graft by the host immune response [33–36].

Pre-clinical studies have generally employed xenogeneic transplantation strategies, injecting human derived cells into heavily immunosuppressed animal hosts, noting minimal immune rejection [13••, 14••, 29, 30•]. However, immunosuppression, particularly of vulnerable heart failure patients is not without risk, and strategies to bypass the host immune response without drug therapy are desirable.

The advent of iPSCs heralded hopes of autologous cell transplantation negating fears of graft rejection; however, progress on this front has been limited by manufacturing and regulatory hurdles [37]. Reprogramming somatic cells into iPSCs and subsequently differentiating these to good manufacturing practice (GMP) grade cardiomyocytes from individual patients is an expensive and time-consuming process, taking up to 6 months and thus not a practical or scalable option at the current time [38].

Allogeneic transplantation of MHC matched iPSCs is a promising alternative, allowing for banking of cryopreserved products and more timely distribution; however, studies using this approach in large animals suggest that some degree of immunosuppression would still be required to avoid rejection [16••, 39, 40]. Acquiring a cell bank that caters to modern, multicultural and genetically diverse societies may also prove to be an extremely challenging task [41, 42].

The most likely way forward appears to be using gene editing technologies. Knockout of human leukocyte antigens may generate ready-to-use immunocompatible cells, and exciting progress has already been made on this front [43–47]. However, further studies exploring the efficacy of transplanting cardiomyocytes derived from these ‘universal donor cells’ are required.

### Engraftment

Aside from the host immune response, factors such as cell delivery technique, engraftment environment and the ability of transplanted cells to physically integrate with the host heart can also greatly affect retention and long-term survival. In fact, it has been reported that less than 10% of injected cells successfully engraft and survive after delivery into the heart [48, 49]. Of these surviving cells, however, graft expansion has been noted, and it is now known that this phenomenon can be attributed to a subset of transplanted cardiomyocytes with intrinsic proliferative capacity [50]. Harnessing this proliferative capacity by transcriptional manipulation may accentuate the potential of PSC-CM therapy to repopulate damaged myocardium. Indeed, it was recently shown that overexpression of cyclin D2, a protein involved in cell cycle regulation, led to significantly increased graft sizes and subsequent improvements in left ventricular function in both small and large animal models [51, 52].

Improving functional integration of transplanted cells with native myocardium has also been explored. Over-expression of N-cadherin, a cell adhesion protein, has been shown to improve engraftment and increase the survival of implanted cells [53]. Importantly, mice receiving these genetically modified cells also had reduced infarct sizes and improved left ventricular function compared to cell-treated controls.

Given transplanted cardiomyocytes die from ischaemia in the first few days following transplantation, creating a more favourable environment by enhancing local vascularization may promote cell survival [54, 55]. This hypothesis was tested in a rodent study in which PSC-CMs were co-transplanted with ready-made microvessels obtained from adipose tissue [56]. Microvessels showed persistence and integration at early and late time points, resulting in increased graft perfusion and survival and leading to improved functional recovery following myocardial infarction.

Creating PSC-CM grafts of sufficient size to support clinically meaningful function is one of the key challenges facing cardiac remuscularization [57]. A combination of generating cardiomyocytes with greater proliferative and integrative potential along with developing a more favourable transplant environment are both important strategies in the pursuit of improving PSC-CM engraftment.

### Arrhythmogenicity

Perhaps the most concerning hurdle to PSC-CM clinical translation is arrhythmogenicity. Ventricular arrhythmias have now been identified post-cell transplantation in several large animal studies [13••, 14••, 16••, 29, 30•]. Though initial reports from small animal experiments proposed this cell type conferred anti-arrhythmic effects, this has been unequivocally refuted in larger animals with similar cardiac size and physiology to humans [28]. In hindsight, the high basal heart rates of rodents (500–600 beats per minute) likely surpassed the maximal contraction rate of transplanted cardiomyocytes, masking the arrhythmogenic potential of these cells.

Even though these arrhythmias are often transient, generally subsiding within a few weeks following cell transplant, if left untreated, heart failure can ensue, and in severe cases sudden cardiac death can result from degeneration of the rhythm into ventricular fibrillation [29, 30•]. Electrophysiological studies performed in animals with sustained engraftment arrhythmia have demonstrated enhanced automaticity at the site of cell engraftment, suggesting the cell grafts behave as an ectopic pacemaker superseding the host’s sinoatrial node [14••, 29].

Although postulated mechanisms underpinning these arrythmias range from factors related to cell preparation, transplantation techniques or host characteristics, the mechanism likely relates to the intrinsic characteristics of input cells. PSC-CM exhibit automatic behaviour with spontaneous contraction in vitro, and this automaticity appears to be retained post transplantation until the graft matures and forms stable electrical connections with the recipient heart in vivo [8, 13••, 29, 58, 59]. Histological assessment has shown greater expression of gap junction proteins such as connexin 43 at later timepoints when engraftment arrhythmias have been shown to subside, supporting the notion that early impairment of electrical coupling between host and graft is important in arrhythmogenesis [13••, 29].

Current differentiation protocols yield relatively immature cardiomyocytes, with calcium handling and electrophysiological properties akin to foetal cardiomyocytes [60, 61]. In addition, heterogenous cell products are common, and though ventricular cardiomyocytes predominate, a mixture of other cells is present including atrial myocytes, cardiac conduction tissue, fibroblasts and endothelial cells [62–64]. This cellular diversity may contribute to arrhythmogenicity, and in particular, the presence of pacemaker cells may be a driving factor in the automatic arrhythmias encountered.

A recent study has demonstrated that pharmacologic therapy can supress engraftment arrhythmias [30•]. Here, a combination drug treatment with amiodarone and ivabradine significantly improved but could not completely eradicate these cardiac rhythm disturbances. This highlights the need to optimise cell products prior to pursuing clinical translation [30•]. Interestingly, blockade of the pacemaker current with ivabradine was shown to have potent effects in reducing the heart rate of pigs in engraftment arrhythmia, again alluding to the potential importance of attenuating cellular automaticity and pacemaker myocyte subpopulations.

Cell dosage may be another important factor in arrhythmogenicity. Initial studies in pigs and non-human primates have delivered cell doses ranging from 750 million to 1 billion cardiomyocytes, though much lower cell doses of 2–120 million cardiomyocytes transplanted into infarcted pig hearts have not result in substantial engraftment arrhythmias, raising questions on whether cell dose may be an important predictor of arrhythmic burden [13••, 14••, 16••, 29, 30•, 51, 65, 66].

Overcoming the arrhythmogenicity of PSC-CMs is paramount to its clinical success, and to date a complete solution remains elusive. It is likely that modification of both cell specific and graft recipient factors will be required. This may include addressing the cellular heterogeneity of current differentiation protocols, enhancing maturity and gap junctions of engrafted cardiomyocytes and conducting dose escalation experiments to identify the optimal PSC-CM transplantation dose.

### Concluding Remarks on Clinical Challenges

Though great promise has been shown, there remain limitations to PSC-CM therapy in its current form and addressing these is the focus of intense worldwide research efforts. Thorough knowledge of cardiomyocyte differentiation strategies and their underlying biology are required to develop cells more fit for widescale clinical use. In the following sections, we shift our attention to explore the basic studies and biology of PSC-CMs, highlighting differentiation and cell production strategies along with techniques to characterize output cells. Understanding these processes may help guide future cell products with robust reparative capacity and minimal adverse effects.

## Cardiac PSC Differentiation Strategies

Knowledge from developmental biology, specifically the induction of mesoderm and cardiac lineages, has guided the many cardiac differentiation strategies currently established for use with pluripotent stem cells. These methods try to recapitulate different stages of embryonic development, with many studies elucidating signalling pathways that lead to primitive streak formation (stage 1), induction and specification of cardiac mesoderm (stage 2) and the expansion of committed cardiac lineage cells (stage 3) (Fig. 1B).

### Growth Factors and Peptides

Initial cardiac PSC differentiation strategies utilised growth factors and peptides involved in key cardiovascular development pathways (Fig. 1B). These include the activin/nodal/transforming growth factor (TGF)-β, Wingless-related integration site (Wnt) and bone morphogenic progenitor (BMP) pathways, and as such the growth factors, Activin A, BMP, basic fibroblast growth factor (bFGF), insulin growth factor (IGF)-1, vascular endothelial growth factor (VEGF), Wnt and Dickkopf-1 (Dkk-1), have all been investigated for cardiomyocyte differentiation [64, 67–73]. Initial studies demonstrated the dose, timing and combination of growth factors are critical for effective cardiomyocyte differentiation. For example, using the endogenous Wnt inhibitor, Dkk-1, at the beginning of culture (day 2–3) suppressed cardiac mesoderm induction, whilst Wnt later inhibition (day 4) promoted cardiac specification [67, 74]. Studies investigating different combinations of growth factors showed the optimal yield of pure cardiomyocytes across multiple PSC cultures was best achieved with serial applications of BMP and Activin A [67].

Techniques to produce specific cardiomyocyte subpopulations of interest have also been explored. Recently, the importance of retinoic acid signalling in determining cardiomyocyte fate has been reported [64, 75–79]. Activation of this pathway commits cardiomyocyte differentiation towards an atrial or pacemaker lineage, whereas inhibition may promote generation of more ventricular myocytes.

Though growth factor and peptide-based differentiation strategies have been favoured in pre-clinical transplantation studies, the feasibility of upscaling cell production using this approach would be cost prohibitive for clinical translation, highlighting the need for alternative approaches [13••, 14••, 28, 80].

### Small Molecule Agonists and Antagonists

Using small-molecule agonists and antagonists is one such alternative strategy that overcomes the burden of cost associated with growth factor-directed differentiation methods (Fig. 1B). Most of these agents harness the important temporal role of Wnt signalling on cardiac differentiation as described above [81, 82]. Though initially relying on endogenous Wnt inhibitors such as Dkk-1, this strategy later evolved to use small-molecules in conjunction with growth-factor and serum-free protocols.

In general, these protocols involve the addition of CHIR99021, a Wnt activator, to PSC culture at the beginning of differentiation (day 0), triggering cardiac mesoderm induction. This is followed by the addition of small-molecule inhibitors of Wnt ligand production (IWPs) at day 3 using insulin-free medium to commit cells to the cardiomyocyte fate [83, 84]. This differentiation strategy proved to be more robust than growth-factor mediated techniques, generating 80–98% cardiomyocyte purity across multiple PSC lines, and has now been adopted as the preferred differentiation protocol for several pre-clinical studies [83–86].

### Refining Cardiac Differentiation Strategies

Attention has now turned to the important refinement of these traditional differentiation protocols, seeking ways to improve expansion, purity and survival of derived cardiomyocytes. An early study demonstrated that heat shock treatment by incubating cardiomyocytes at 43 ^○^C for 30 min improved cell survival and engraftment [25]. Given the effectiveness of the extracellular matrix (ECM) to support PSC cultures, the addition of Matrigel, a commercially available ECM preparation, was tested and shown to substantially increase both cardiomyocyte purity and survival [71, 87, 88]. Furthermore, the addition of pro-survival factors (anti-apoptotic Bcl-XL, mitochondrial membrane blocker cyclosporine A, IGF-1 and caspase inhibitor, ZVAD-fmk) as well as using insulin-free medium or ROCK inhibitor (Y27632) has been shown to generate a higher proportion and improve survival of cardiomyocytes [69, 84]. Other studies have explored the effect of cellular media, cell–cell contact and physical separation to improve cardiomyocyte yield and purity [72, 89–91].

One of the major advancements in cardiomyocyte purification was the use of metabolic selection [92]. This method takes advantage of the differences of glucose and lactate metabolism between cardiomyocytes and non-cardiomyocytes. The culturing of PSC derivatives in glucose-depleted and lactate-abundant conditions favoured the survival of cardiomyocytes, with ≥ 95% purity.

## Scalability and Production of PSC-CM

Alongside the purity of PSC-CM, it is also important to consider the most efficient production method given that potentially billions of cardiomyocytes will be required for clinical trials. Early protocols used embryoid bodies in serum-dependent systems; however, these produced inconsistent results and more concerningly low cardiomyocyte yield [93]. Protocols later evolved to a monolayer-based, serum-free system which drastically improved the efficiency of cardiac differentiation. However, the scalability and reproducibility of myocyte yields using these protocols better suited small-scale applications such as disease modelling, drug screening, in vitro studies and small animal studies [94–102]. More recent advancements have come with bioreactor technology, using 3D cultures and stirred tank bioreactors at large volumes to generate billions of PSC-CM with small molecule directed differentiation [103, 104]. When combined with thermoresponsive nanobridges, which allows passaging and dissociation without the need of enzymes or small molecules, cardiomyocyte survival and expansion is drastically improved [105, 106]. As such, bioreactor technology appears a promising manufacturing platform for PSC-CM expansion as the field moves forward to clinical translation.

## Characterization of PSC-CM

Though the differentiation strategies to procure pure PSC-CM have improved over time, there is still a considerable degree of heterogeneity of both myocyte and non-myocyte populations with current differentiation strategies [107•]. As highlighted earlier, this cellular diversity may contribute to the therapeutic limitations arising post transplantation, and as such, there is a need to correctly identify cardiomyocyte sub-populations of interest for inclusion or exclusion.

Considerable effort has now been aimed at sub-population characterization by examining a range of parameters including cell morphology, surface marker expression, gene and protein expression, distribution of ion channels and electrophysiological properties (Table 2).

**Table 2:** Makers for characterizing myocytes and non-myocytes derived from PSC cultures

**Cell type**	**Surface marker expression by flow cytometry**	**Gene marker expression by PCR, bulk and/or sRNA-seq**	**Protein marker expression by immunohistochemistry**	**Electrophysiological properties**	**References**
**Myocytes**					
Pan/ventricular	cTnT^+^, SIRPA^+^, NKX2-5^+^, VCAM^+^, MLC2V^+^, MYL2^+^, CD77^+^CD200^−^, HEY2^+^	*cTnT*, *NKX2*-5, *MLC2V*, *MYL2*, *IRX4*, *MEF2C*, *MYH6*, *MYH7*, *GJA1*, *PDLIM1*, *VTN*, *LPL*, *HEY2*	cTnT, MLC2V, MYL2, NKX2-5, MYH7, α-actinin, VCAM	Fast upstroke velocity (> 10 V/s), longer AP duration, plateau phase	[15••, 72, 87, 104, 107•, 108, 110, 115•, 116–119]
Immature ventricular	-	*KDR*, *ISL*, *GATA4*, *TNNI1*; Churko et al. [121•] noted the following genes were enriched in early-stage cardiomyocytes, i.e. day 14: *VCAM1*, *APOE*, *NPPA*, *NPPB*, *MYH6*, *COL2A1*, *HMGA1*, *S100A10*, *ATF4*, *RBFOX2*, *HMGA2*, *CLDN6*	-	Slow upstroke velocity (< 10 V/s), slow beating rate (50 bpm)	[86, 104, 118, 119]
Mature ventricular	CD36^+^SIRPA^+^LDLR^+^	*MYH7*, *HEY2*; Churko et al. [121•] noted the following genes were enriched in day 45 cardiomyocytes: *HEY2*, *FHL2*, *CAV1*, *SERPINI1*, *MYL2*, *A2M*, *LGALS3BP*, *TMEM173*, *SYNE2*, *NFIA*, *HOPX*; Funakoshi et al. [113•] classified mature cells with the following highly expressed genes: FAO: *CD36, FABP3*, *ACSL1;* Mitochondrial: *CKMT2*, *COX6A2;* Muscle function: *DES*, *MYBPC3*, *ACTN2* and further classified cell types as non-proliferating, non-stressed (*CD36*, *LDLR*), stressed (*ATF5*, *TRIB3*), proliferating (*MKI167*, *FOXM1*) and cardiac fibroblasts (*FN1*, *COL3A1*)	-	-	[104, 110, 118]
Atrial	MLC2A^+^	*ACTA2*, *ATP2A2*, *CACNA1D*, *GJA5* (*CX40*), *HEY1*, *KCNA5*, *KCNK3*, *KCNJ3*, *MLC2A*, *MYH6*, *MYLK*, *MYL7*, *NR2F1*, *NR2F2*, *NPPA*, *SLN*, *TBX5*, *RBP1*	MLC2A	Triangular morphology	[77, 87, 110, 115•, 116–119]
Pacemaker	-	Transcription factor: *SHOX2*, *ISL1*, *TBX3*, *TBX5*, *TBX18*; ion channel: *HCN1*, *HCN4*, *KCNJ3*, *SCN5A*	TBX3	Fast spontaneous firing rates, slow maximum upstroke velocity, small AP amplitudes, short AP durations; faster beating rates	[104, 115•, 117, 119]
SAN pacemaker	-	*ISL1*, *SHOX2*, *TBX3*, *TBX18*	SHOX2	-	[87, 104, 115•]
AVN pacemaker	-	*MSX2*, *TBX2*	-	-	[115•]
**Non-myocytes**					
Pluripotency markers	OCT4^+^, SOX2^+^, TRA160^+^, SSEA-4^+^	*POU5F1*, *DNMT3B*, *NANOG*	OCT4, NANOG, TRA-1–80, SSEA-4	N/A	[15••, 82, 87, 104]
Endoderm	-	*SOX17*, *FOXA2*	-	N/A	[104]
Mesoderm	Flk-1^+^PdgfR-α^+^, CD235a^+^ (ventricular mesoderm), RALDH2^+^ (atrial mesoderm), CD13^+^ (early cardiac mesoderm)	*EOMES*, *T*, *MESP1*, *MIXL1*	-	N/A	[66, 87, 104, 118–120]
Fibroblast	CD90^+^	*CD90*	P4HB, TE-7	N/A	[15••, 87, 104, 108, 119]
Epithelial	-	*-*	PanCK	N/A	[15••]
Endothelial	VE-cad^+^, PECAM^+^, CD31^+^, CD34^+^	*CDH5*, *CD34*, *PECAM*, *TAL1*	vWF, CD31	N/A	[15••, 72, 104, 107•, 108, 115•, 121•]
Vascular smooth muscle	CNN1^+^	*ACTA2*, *CNN1*, *MYH11*, *TAGLIN2*	αSMA	N/A	[71, 86, 104, 107•]

## Markers to Distinguish Cardiomyocytes from Non-myocytes

From a manufacturing perspective, identifying markers that facilitate the isolation of pure cardiomyocytes is critical for quality control. Gene expression profiles by real-time reverse-transcription polymerase chain reaction (RT-PCR) are commonly used at different timepoints during cell production to confirm validity of differentiation protocols. Downregulation of pluripotency markers (*POU5F1*) concurrent with the upregulation of mesodermal markers (*MESP1*, *T*, *MIXL1*) signify the cell committing to mesoderm induction in the first few days of differentiated cultures (day 2), followed by the expression of early cardiomyocyte markers, (*KDR*, *ISL* and *GATA4*) mid-culture (days 5–6), and later the expression of markers such as *NKX2*-5, *TBX5* and *MEF2C* which signify a cardiomyocyte fate [89].

At the completion of differentiation, cardiac troponin T positivity has become the gold standard to deduce cardiomyocyte purity; however, more recently, additional cardiomyocyte markers such as signal regulatory protein alpha (SIRPA) and vascular cell adhesion protein (VCAM) have also been uncovered further improving reliability of cardiomyocyte detection [73, 108, 109].

Identification of non-cardiomyocytes is of equal importance in assessing differentiation purity. Marker signatures for several non-myocyte cell types are now known, including endothelial cells (VE-cadherin, CD31, CD34), vascular smooth muscle cells (transgelin, calponin), mesenchymal/fibroblast-like cells (CD90, TE-7), endodermal cells (SOX17, FOXA2, EpCAM) and pan-mesodermal cells (EOMES) [15••, 73, 107•, 110, 111].

Hence, both gene and surface marker expression can be used as important quality control tools in the process of manufacturing pure and reproducible clinical grade PSC-CMs.

### Cardiomyocyte Maturity Markers

Current differentiation protocols yield phenotypically immature cardiomyocytes, one of the critical issues contributing to arrhythmogenicity post transplantation [112]. These immature cells have foetal cardiomyocyte features, including altered calcium handling, weak contractions and poor subcellular organization and structural alignment [112]. Contrastingly, ‘more’ mature cardiomyocytes derived from PSC cultures are better reflective of adult human cardiomyocytes possessing increased cell size, greater proportion of multinucleated cells, more compact myofibril density, alignment and organization, faster calcium handling and stronger contractile performance [62, 112, 113•].

Though prolonged culture and other techniques to hasten maturation have been described, such processes would balloon production costs for widescale clinical applications [62, 112, 114, 115•, 116]. Thus, understanding and potentially altering important genes involved in cardiomyocyte maturation may be of great benefit moving forward into clinical translation. Single cell ribonucleic acid sequencing (scRNA-seq) experiments have broadened our knowledge in this area, detecting genes expressed earlier (*TNNI1*, *MYH6*) and later (*NKX2-5*, *MYH7*, *MYL2*, *TTN*, *TNNI3*, *MYL2*) in embryonic heart development to identify immature and mature cardiomyocytes [107•]. Metabolic gene signatures associated with cardiomyocyte maturity have also been elucidated, along with potential surface markers denoting mature cardiomyocytes [113•].

### Markers to Distinguish Ventricular Cardiomyocytes from Atrial and Pacemaker-Like Cells

Also of particular importance to the arrhythmogenicity of PSC-CMs is cellular heterogeneity, with current differentiation protocols producing atrial and pacemaker cells in addition to the more electrically quiescent ventricular cardiomyocytes.

Thus, it is advantageous to distinguish cardiomyocyte sub-populations from one another, with ventricular cardiomyocytes being the desired subpopulation for therapeutic benefit in clinical indications such as heart failure. NKX2-5 is considered a useful marker to differentiate atrial and ventricular cardiomyocytes from sinoatrial node (SAN) pacemaker cardiomyocytes given that its expression is absent in the second heart field lineage, from where SAN cells are thought to arise [117]. The surface marker signature NKX2-5^−^SIRPA^+^CD90^−^ has been shown to efficiently isolate a highly enriched SAN-like pacemaker population, whereas a CD77^+^CD200^−^ signature has been shown to effectively isolate > 97% troponin-positive ventricular cells [118, 119].

These surface marker signatures carry the great advantage of allowing isolation of desired subpopulations through fluorescence-activated cell sorting (FACS). However, though this may be a suitable strategy to generate pure cardiomyocyte subtypes for use in in vitro or small animal experiments, it may not meet the needs of clinical translation due to excessive time and cost constraints, along with substantial reductions in overall cell yield.

Gene expression profiles obtained by RT-PCR have also been routinely used to characterize PSC-CM sub-populations as either atrial (*NPPA*, *GJA5*, *KCNA5*, *SLN*), ventricular (*MLC2V*, *IRX4*) or nodal (*TBX18*) [64, 89, 120]. Both bulk and scRNA-seq experiments have revealed that strict categorization of cell types to ventricular, atrial or pacemaker-like cardiomyocytes by their gene expression profile alone has limitations given that gene expression may be transient and/or more reflective of cardiomyocyte maturation than subpopulation determination [121•]. This should be kept in mind for the characterization of PSC-CM fated for clinical trials.

## Conclusion

Regenerative medicine in the cardiovascular field is at a particularly exciting stage. Learning from lessons of the adult stem cell field, new robust PSC-CM clinical trials have commenced with several more working through the regulatory stages. Barriers to ultimate widescale clinical translation remain. However, this is normal for any new cutting-edge therapeutic technology. Iterative and incremental learning will continue through to phase III/IV clinical trials and beyond. Further understanding of cardiovascular developmental biology and characterization of PSC derivatives may help streamline current cardiac differentiation strategies, cultivating a cell product capable of robust engraftment and infarct repair whilst avoiding potential immuno- and arrhythmogenicity. A worldwide, cross-disciplinary approach is needed to progress our biological understanding of PSC-derived cardiac lineages along with their therapeutic applications and clinical effects.
